# Cyanobacterial nitrogenases: phylogenetic diversity, regulation and functional predictions

**DOI:** 10.1590/1678-4685-GMB-2016-0050

**Published:** 2017-03-20

**Authors:** Alberto A. Esteves-Ferreira, João Henrique Frota Cavalcanti, Marcelo Gomes Marçal Vieira Vaz, Luna V. Alvarenga, Adriano Nunes-Nesi, Wagner L. Araújo

**Affiliations:** 1 Departamento de Biologia Vegetal, Universidade Federal de Viçosa, Viçosa, MG, Brazil; 2 Max-Planck-partner group at the Departamento de Biologia Vegetal, Universidade Federal de Viçosa, Viçosa, MG, Brazil

**Keywords:** Cyanobacteria, evolution, hydrogen, nitrogen fixation, molecular phylogeny

## Abstract

Cyanobacteria is a remarkable group of prokaryotic photosynthetic microorganisms, with several genera capable of fixing atmospheric nitrogen (N_2_) and presenting a wide range of morphologies. Although the nitrogenase complex is not present in all cyanobacterial taxa, it is spread across several cyanobacterial strains. The nitrogenase complex has also a high theoretical potential for biofuel production, since H_2_ is a by-product produced during N_2_ fixation. In this review we discuss the significance of a relatively wide variety of cell morphologies and metabolic strategies that allow spatial and temporal separation of N_2_ fixation from photosynthesis in cyanobacteria. Phylogenetic reconstructions based on 16S rRNA and *nif*D gene sequences shed light on the evolutionary history of the two genes. Our results demonstrated that (*i*) sequences of genes involved in nitrogen fixation (*nif*D) from several morphologically distinct strains of cyanobacteria are grouped in similarity with their morphology classification and phylogeny, and (*ii*) *nif*D genes from heterocytous strains share a common ancestor. By using this data we also discuss the evolutionary importance of processes such as horizontal gene transfer and genetic duplication for nitrogenase evolution and diversification. Finally, we discuss the importance of H_2_ synthesis in cyanobacteria, as well as strategies and challenges to improve cyanobacterial H_2_ production.

## Introduction

**Figure 1:**
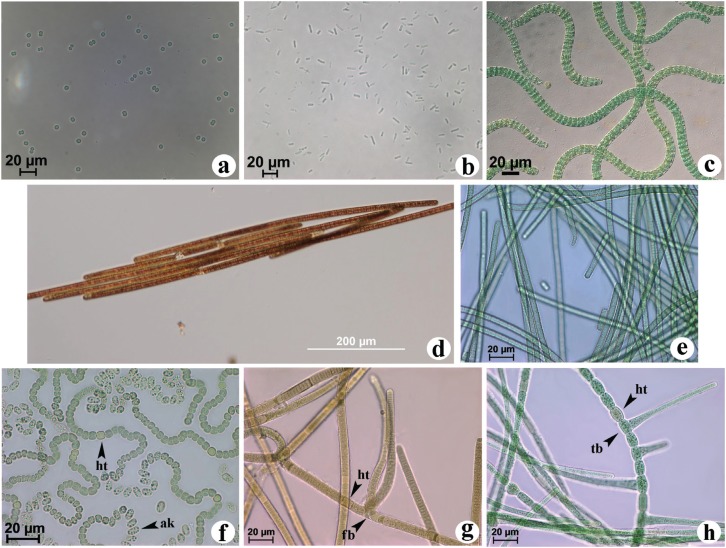
Natural morphological variation within cyanobacterial genera. Unicellular strains: (a) *Synechocystis* sp. PCC6803 and (b) *Synechococcus elongatus* PCC4972. Non-heterocytous strains: (c) *Arthrospira maxima,* (d) *Trichodesmium* sp. and (e) *Phormidium* sp. CCM-UFV034. False branching or non-branching heterocytous strains: (f) *Nostoc* sp. CCM-UFV028 and (g) *Brasilonema octagenarum* CCM-UFVE1. True-branching heterocytous strain: (h) *Stigonema* sp. CCM-UFV036. *Synechocystis* sp. (a) and *Arthrospira maxima* (c) are unable to perform N_2_ fixation, whereas the strains shown in b and e present a temporal separation of metabolism: photosynthetic CO_2_ fixation is performed in the light while N_2_ fixation occurs during darkness. *Trichodesmium* sp. (d) is the unique non-heterocytous cyanobacterium that shown N_2_ fixation under light conditions. Conversely, in other strains (f, g, and h), there is a spatial separation of metabolism, with N_2_ fixation occurring in heterocytes (ht). Abbreviations: (ak) akinetes; (fb) false branching; (tb) true branching. The picture of *Arthrospira maxima* (c) was kindly provided by the Culture Collection of Autotrophic Organisms (CCALA), http://ccala.butbn.cas.cz and the picture of *Trichodesmium* sp. (d) by Prof. Ondøej Práil, Institute of Microbiology, Czech Academy of Sciences, Czech Republic. The other pictures are from strains kept at Collection of Cyanobacteria and Microalgae from Universidade Federal de Viçosa (CCM-UFV).

Cyanobacteria is a biochemically and morphologically diverse group of gram-negative bacteria capable of perform oxygenic photosynthesis (Figure 1). These microorganisms are observed in fresh water, marine and terrestrial habitats, being the major primary producers in these ecosystems (Hess, 2011). Fossil records indicate that cyanobacteria have been extant for at least 2.5 billion years (Knoll, 2008). Additionally, it is likely that the ancestors of cyanobacteria played a key role in the formation of atmospheric oxygen (O_2_) (Hackenberg *et al.*, 2011) and are also believed to have evolved into the present-day chloroplasts of green algae and plants (Koonin, 2010; Keeling, 2013). Cyanobacterial metabolic plasticity appears to have permitted these organisms to withstand the challenges of evolutionary environmental changes and has enabled them to survive and colonize diverse habitats (Steinhauser *et al.*, 2012). Indeed, these organisms exhibit enormous diversity in terms of their habitats, morphology, physiology, and metabolism (Beck *et al.*, 2012). These microorganisms display a relatively wide variety of morphologies, such as unicellular, non-heterocytous and heterocytous filamentous strains with the latter showing different types of cells (heterocytes and akinetes) (Schirrmeister *et al.*, 2013) (Figure 1a-h). Heterocytous strains are able to form differentiated cells, specialised in nitrogen (N_2_) fixation, the heterocytes, and spore-like resting cells, the akinetes (Figure 1f-h). However, a number of unicellular (Figure 1a-b) and non-heterocytous (Figure 1c-e) strains are also able to perform N_2_ fixation under certain conditions, despite the absence of specialised cells (Berman-Frank *et al.*, 2003).

**Figure 2:**
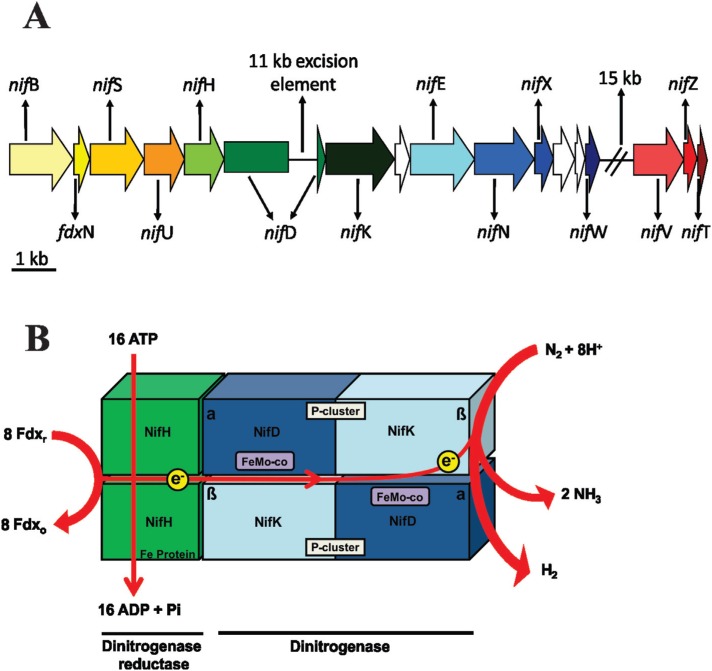
Nitrogenase gene structure. (A) Map illustrating two of the *nif* gene clusters present in *Anabaena variabilis* ATCC29413. The operons are represented using different colours: yellow (*nif*B-*fdx*N-*nif*SU), green (*nif*HDK), blue (*nif*ENXW) and red (*nif*VZT). White arrows indicate genes which encode proteins with unknown functions. The 11-kb excision element observed in the *nif*HDK operon is not present in the chromosomes of heterocytes. *nif*VZT is part of a second *nif* gene cluster. (B) Nitrogenase structure and biochemical activity. Dinitrogenase reductase is a homodimer (2×30 kDa) with one [4Fe-4S]-cluster (Fe protein) encoded by *nif*H, whereas dinitrogenase is a FeMo-protein encoded by the genes *nif*D (α-subunit) and *nif*K (β-subunit) and organized in a α2β2 tetramer of 240 kDa associated with two FeMo-cofactors (FeMo-co) and two P-clusters. Notably, there are three types of dinitrogenases normally found in cyanobacterial nitrogenases, which vary depending on the metal content. Thus, type 1 contains molybdenum (Mo), type 2 contains vanadium (V), and type 3 iron (Fe). The reduction of nitrogen (N_2_) to ammonia (NH_3_) requires metabolic energy in the form of ATP, and in this context two ATP molecules are used for each electron transferred from the dinitrogenase reductase to dinitrogenase. Consequently, the reaction requires a total of 16 ATP molecules until the dinitrogenase has accumulated enough electrons to reduce N_2_ to NH_3_. In addition, this reaction is accompanied by the reduction of two protons (H^+^) to one hydrogen molecule (H_2_).

Occurrence of nitrogen fixation (*nif*) gene clusters has been reported in several organisms. However, all known N_2_-fixing organisms are prokaryotes and thus the ability to fix N_2_ is widely, though paraphyletically, distributed across bacterial and archaeal domains (Staley and Reysenbach, 2002; Raymond *et al.*, 2004). Additionally, *nif* genes have been identified in 21 out of the 44 sequenced cyanobacterial genomes thus far, including terrestrial and marine strains (Boyd and Peters, 2013). They are organized in distinct operons namely *nif*B-*fdx*N-*nif*SU, *nif*HDK, *nif*ENXW, and *nif*VZT (Figure 2A). Interestingly, in *Anabaena* spp. there is an 11-kb excision element in the *nif*HDK operon, which is removed from the chromosome during the differentiation of vegetative cells to heterocytes, allowing the transcription of the complete operon (Golden and Wiest, 1988; Brusca *et al.*, 1990). Moreover, *nif*VZT is present in a separate *nif* gene cluster (Figure 2A) (Pratte and Thiel, 2014). The intrinsic capacity for N_2_ fixation in cyanobacteria is related only to the nitrogenase enzyme system, with the molybdenum nitrogenase (Mo-nitrogenase) being the most studied nitrogenase (Betancourt *et al.*, 2008) (Figure 2B). This enzymatic complex comprises approximately 10% of the total cellular protein in many diazotrophs, requesting 16 ATPs per N_2_ fixed (Simpson and Burris, 1984; Berman-Frank *et al.*, 2003), and catalyzes the synthesis of approximately half of all of the fixed N_2_ on Earth nowadays (Falkowski, 1997). In addition, nitrogenase also catalyzes the production of hydrogen (H_2_) as a by-product of N_2_ fixation (Seefeldt *et al.*, 2009; Bothe *et al.*, 2010). Nitrogenase enzyme consists of two parts: the dinitrogenase, a FeMo-protein encoded by the genes *nif*D (α-subunit) and *nif*K (β-subunit), which is organized in a α2β2 tetramer of 240 kDa associated with two FeMo-cofactors (FeMo-co) and two P-clusters; and the dinitrogenase reductase, a homodimer (2 × 30 kDa) with one [4Fe-4S]-cluster (Fe Protein) encoded by *nif*H (Figure 2B) (Orme-Johnson, 1992; Böhme, 1998).

Although the function of the proteins encoded by the other *nif* genes in *Anabaena variabilis* ATCC29413 (a model filamentous heterocytous strain for physiological studies) remains elusive, possible functions were inferred by analysing *nif* genes already described in other diazotrophic bacteria (Böhme, 1998). NifE and NifN is a heterotetrameric complex similar to NifD and NifK respectively, and seems to act as scaffolds for FeMo-co assembly (Fani *et al.*, 2000). NifV is a homocitrate synthetase which provides homocitrate for the FeMo-co biosynthesis (Zheng *et al.*, 1997; Mayer *et al.*, 2002). NifS and NifU are involved in iron and sulphur mobilization, and in the assembly of [4Fe-4S] clusters (Rubio and Ludden, 2008). Thus, these [4Fe-4S] clusters are transferred to NifB, and converted into NifB-cofactor, a precursor for the biosynthesis of FeMo-co (Curatti *et al.*, 2007). NifX is able to bind precursors of the FeMo-co, being a transient reservoir for these molecules (Hernandez *et al.*, 2007). NifW is not directly involved in the FeMo-protein assembly, but associate with it under aerobic conditions, being part of an O_2_ protection system (Kim and Burgess, 1996). NifZ is important for P-cluster maturation (Hu *et al.*, 2007) and the function of NifT remains unclear (Thiel and Pratte, 2014).

Cyanobacteria play major environmental and economic roles, including global primary productivity (Paerl, 2012), potential uses in renewable energy, commonly referred to as ‘third-generation fuels’, (Dismukes *et al.*, 2008; Hu *et al.*, 2008), and are also an immense source of valuable natural products with biotechnological applications (Pulz and Gross, 2004; Rastogi *et al.*, 2010; Wijffels *et al.*, 2013). This photosynthetic group of prokaryotic organisms can therefore be developed as a highly productive microbial cell factory that can harvest solar energy and capture carbon dioxide from the atmosphere, converting it into both biofuels and several useful products (Parmar *et al.*, 2011). The high photosynthesis capabilities of cyanobacteria allow them to convert up to 10% of the received solar energy into biomass, in comparison with 5-6% energy conversion registered for C4 crops such as maize and sugar cane, and 5% for algae (Parmar *et al.*, 2011; Jones and Mayfield, 2012; Ogawa and Sonoike, 2015). Accordingly, cyanobacteria are advantageous organisms for use in industrial applications because they exhibit rapid cell growth, have simple nutrient requirements (mainly water, sunlight and CO_2_) (Ruffing, 2011) and are naturally transformable, thus presenting the potential to be genetically engineered (Heidorn *et al.*, 2011; Ruffing, 2011; Wilde and Dienst, 2011). Due to their natural morphological (Figure 1a-h) and physiological diversity coupled with their capacity of growing in a variety of environments, even in areas that are inappropriate for agriculture, there is a growing interest to understand cyanobacterial strains (Machado and Atsumi, 2012). However, despite their promise, different biotechnical, environmental and economic bottlenecks have to be overcome before cyanobacteria can become industrial large scale microorganisms (Parmar *et al.*, 2011; Savage, 2011). One such challenge clearly involves research towards the application of cyanobacteria for the production of alternative biofuel sources, as molecular techniques for metabolic genetic engineering are currently available and in use (Wijffels *et al.*, 2013). Thus, although research towards the application of cyanobacteria for biofuel production has been mainly focused on strains belonging to the genera *Synechocystis* and *Synechococcus*, with much smaller additional efforts being carried out in N_2_-fixing strains such as *Anabaena, Nostoc* and some *Cyanothece*, despite nitrogenase being a promising candidate for photobiological hydrogen (H_2_) production.

In this study, we asked whether cyanobacterial nitrogenase complex evolution is congruent with the morphological and 16S rRNA diversity. Due to the higher availability and quality of the *nif*D nucleotides sequences in comparison to the one for *nif*K and *nif*H, we selected the first gene to perform the phylogenetic analysis. For this purpose, phylogenetic reconstructions based on partial sequences of *nif*D and 16S rRNA gene sequence were perfomed. The results presented here are a compilation of robust phylogenetic analyses performed by us including a vast number of cyanobacterial strains. Then, we discuss our phylogenetic findings comparing it with previously published data on nitrogenase phylogenetic evolution, placing special attention to nitrogenase and the role of this enzyme during cyanobacterial evolution. Finally, we discuss the regulation of different strategies used by cyanobacteria to avoid nitrogenase inactivation and degradation by O_2_, providing an update on technologies and molecular tools that have been developed to allow increased cyanobacterial hydrogen production.

## Can N_2_ fixation strategies be associated with cyanobacterial morphology?

The expression of *nif* genes is controlled by the carbon-nitrogen (C/N) balance and cellular redox status in cyanobacteria (Valladares *et al.*, 1999), with 2-oxoglutarate being a signal molecule of the cellular nitrogen levels (Lee *et al.*, 1999; Zhao *et al.*, 2010). Ammonium, as the most reduced inorganic form of nitrogen, is the preferred source of nitrogen for cyanobacteria. Hence, when present in the environment, it represses indirectly the expression of *nif* genes by blocking the transcription of NtcA, a transcriptional activator associated with global nitrogen control in cyanobacteria (Herrero *et al.*, 2001). Then, in the presence of nitrogen sources other than ammonium, or under nitrogen starvation, NtcA activates the transcription of a set of genes by binding to the target consensus nucleotide sequence GTAN8TAC present in the promoter region (Luque *et al.*, 1994). These genes include not only the *nif*HDK operon encoding the nitrogenase complex (Muro-Pastor *et al.*, 2002), but also genes involved in heterocyte development (Flores and Herrero, 2005); mobilization of stored nitrogen (phycobilisome) (Luque *et al.*, 2001), assimilation of ammonium via the GS/GOGAT (glutamine synthetase/glutamine oxoglutarate aminotransferase) cycle (Muro-Pastor *et al.*, 1996; Reyes *et al.*, 1997; Vázquez-Bermúdez *et al.*, 2002), sensing and control of cellular nitrogen homeostatsis by the PII protein (García-Domínguez and Florencio, 1997) and NtcA itself (Alfonso *et al.*, 2001; Paz-Yepes *et al.*, 2003). In strains unable to fix N_2_, NtcA activates the uptake of nitrogen sources such as nitrate, urea, and ammonium (Suzuki *et al.*, 1995; Valladares *et al.*, 2002; Flores *et al.*, 2005; Paz-Yepes *et al.*, 2007).

As observed for the activity of nitrogenase, it has been demonstrated that levels of *nif* transcripts and the biosynthesis of different subunities of the nitrogenase complex are very sensitive to O_2_ (Fay, 1992; Staal *et al.*, 2007; Steunou *et al.*, 2008), most likely to avoid energy losses associated with the degradation of this enzyme under high levels of O_2_. Thus, to cope with the production of O_2_ inside their own cells by photosynthesis, which provides energy for all cellular processes, including N_2_ fixation, cyanobacteria have evolved strategies that protect nitrogenase complex from O_2_. Many cyanobacterial strains reconcile nitrogenase activity with photosynthesis (Bergman *et al.*, 1997; Berman-Frank *et al.*, 2003) through spatial and/or temporal separation of these two incompatible metabolic processes (Stal, 2008; Flores and Herrero, 2010; Stal *et al.*, 2010).

Many filamentous cyanobacteria solve the issue by cell differentiation. Thus, in heterocytous cyanobacteria, under aerobic growth conditions, O_2_ evolution and CO_2_ fixation (photosynthesis) is performed in vegetative cells, whereas nitrogenase catalyses N_2_-fixation in specialised cells called heterocytes (Figure 1f-h) (Stal, 2008; Cardona *et al.*, 2009; Stal *et al.*, 2010). These specialised cells differentiate from vegetative cells 12 to 20 h after combined nitrogen sources are removed from the medium, which leads to extensive metabolic changes (Ow *et al.*, 2008). To protect the nitrogenase from O_2_, the photosystem II (PSII) is largely degraded in heterocytes, and because of that, these cells cannot perform the photosynthetic water-splitting reaction, which is associated with an improved respiration rate and the synthesis of a glycolipid layer in the celluar envelope (Murry and Wolk, 1989; Cardona *et al.*, 2009). The synthesis of a bilayered polysaccharide and glycolipid envelope seems to retard the diffusion of gases, which, combined with changes in the photosynthetic apparatus, results in a microoxic environment, allowing nitrogenase activity during the day (Walsby, 1985, 2007). Additionally, these cells are unable to fix CO_2_ photosynthetically (Stal, 2008). To cope with the absence of energy production, vegetative cells provide photosynthetically fixed carbon to the heterocytes, most likely in the form of carbon exported as sucrose. In turn, the heterocytes provide nitrogen, most likely as glutamine formed via the ammonia generated by N_2_ fixation and the action of glutamine synthetase (GS) (Curatti *et al.*, 2002; Burnat *et al.*, 2014). This connection to vegetative cells occurs through a pore which is equipped with microplasmodesma (Böhme, 1998). Additionally, the levels of GS in heterocytes are very high to prevent the inhibition of nitrogenase by ammonium accumulation (Renström-Keliner *et al.*, 1990). Some heterocytous cyanobacteria, such as *Anabaena variabilis* ATCC29413, are able to synthesise a different Mo-dependent nitrogenase (Nif2) in vegetative cells (Thiel *et al.*, 1995; Thiel and Pratte, 2001). This enzyme is synthesized only under anoxic conditions, shortly after nitrogen depletion, and long before heterocytes form (Schrautemeier *et al.*, 1995; Thiel *et al.*, 1997). Additionally, Nif2 is also found in vegetative cells of non-heterocytous species (Berman-Frank *et al.*, 2003). Curiously, the gene *fdxH2,* that is part of the *nif*2 cluster in *A. variabilis* ATCC29413, has more residues in common with the sequence of *fdxH* of the non-heterocytous filamentous cyanobacteria *Plectonema boryanum* PCC73110 (Schrautemeier *et al.*, 1995). Indeed, it seems clear that there is a relation among *nif*2 cluster and *nif* sequences cluster in filamentous non-heterocytous strains (Oscillatoriales and Pseudanabaenales orders), and it is reasonable to assume that the divergence of it might have occurred prior to the heterocytous cell appearance.

Although many unicellular and non-heterocytous cyanobacterial strains can fix N_2_ (Figure 1a-e), the vast majority of them can do this only under anaerobic conditions or, rather, under conditions of decreased O_2_ tension. It has been demonstrated that to fix N_2_, several of these strains have evolved a temporal separation of these two incompatible reactions, with photosynthetic CO_2_ fixation being performed in the light and N_2_ fixation occurring in darkness (Berman-Frank *et al.*, 2007). Amongst these strains, the non-heterocytous filamentous strains *Symploca* and *Lyngbya majuscula* and the unicellular strains *Gloeothece* and *Cyanothece* are worthy of note (Colón-López *et al.*, 1997; Stal, 2008). In these types of cyanobacteria, nitrogenase is typically present in all cells, and a high nitrogenase activity coincides with high respiration rates, with a time difference of 12 h from the peak of photosynthetic activity (Berman-Frank *et al.*, 2003). This pattern is also reflected at the transcriptional level, being observed under either continuous light or darkness, implicating circadian control of these processes (Colón-López *et al.*, 1997; Steunou *et al.*, 2006). In *Synechococcus* an accumulation of *ntc*A transcripts is observed during the day, indicating an insufficiency of fixed N_2_ and promoting an accumulation of *nif* transcripts during the evening, when the net oxygen evolution is low or negative (Steunou *et al.*, 2008).

One conspicuous feature is observed for the marine non-heterocytous filamentous genus *Trichodesmium* (Figure 1d) (Lin *et al.*, 1998). Unlike all other non-heterocytous species of cyanobacteria, in this species, the enzyme nitrogenase is compartmentalised in a fraction of cells called diazocytes (typically between 10 and 20% of the total number of cells) that are often arranged consecutively along the trichome (Lin *et al.*, 1998; Berman-Frank *et al.*, 2001; Rodriguez and Ho, 2014). Diazocytes are structurally different from vegetative cell, since they have a less-granulated aspect. This appearance is related with a decrease in cyanophycin, aerotopes and polyphosphate granules, and an increase in the internal membranes (Fredriksson and Bergman, 1997). These structural changes and the expression of nitrogenase happens in an interval between 8 and 27 hours (Sandh *et al.*, 2012). However, different from heterocytes, diazocytes remained able to perform cell division (Fredriksson and Bergman, 1995). Immunological analyses have revealed the presence of nitrogenase only in diazocytes (Berman-Frank *et al.*, 2001), although few studies propose that almost all cells of *Trichodesmium* are capable of synthesising nitrogenase (Ohki and Taniuchi, 2009). The organisation of the *nif* operon observed in *Trichodesmium* is quite similar with the observed in heterocytous cyanobacteria (Bergman *et al.*, 2013), and contrary to other non-heterocytous cyanobacteria, this genus performs nitrogen fixation during the light period (mid-day), linking both spatial and temporal strategies to improve the efficiency of these processes (Berman-Frank *et al.*, 2001). At mid-day, photosynthesis is down-regulated (Finzi-Hart *et al.*, 2009), and respiration, Mehler reaction, and the pentose phosphate pathway are intensified, decreasing the net O_2_ evolution and providing reducing power (NADPH) for N_2_ fixation, respectively (Sandh *et al.*, 2011). Finally, the ability to fix N_2_ observed in *Trichodesmium* strains was lost in *Arthrospira* (*Spirulina*) (Figure 1c) (Larsson *et al.*, 2011), and although nitrogen fixation has been shown by *Lyngbya* species (Lundgren *et al.*, 2003), a recent study showed the absence of nitrogenase genes in the genome of *Lyngbya majuscula* 3L (Jones *et al.*, 2011)

Phylogenetic analyses were performed based on 16S rRNA and *nif*D gene nucleotides sequences retrieved from National Center for Biotechnology Information (NCBI), GenBank database. Sequences were selected taking into account cyanobacterial taxa from different morphological types. Additionally, for *nif*D gene, sequences from non-cyanobacterial strains, which also belong to the Bacteria domain, were selected. The nucleotide of *nif*D and 16S rRNA sequences retrieved from GenBank were aligned separately using the Muscle algorithm (Edgar, 2004) provided in MEGA 5.0 (Tamura *et al.*, 2011). A total of 39 and 54 sequences were used for *nif*D and 16S rRNA phylogenetic analyses, respectively. For *nif*D gene sequences, a matrix with 6,904 base pair lenght was obtained and a matrix with 1,463 base pair lenght was obtained for 16S rRNA sequences. Optimal evolutionary models were selected using MrModelTest 2.3 (Nylander, 2004) under the Akaike information criterion (AIC). Phylogenetic trees were reconstructed using the maximum-likelihood (ML) and Bayesian methods. For Bayesian analysis, the trees were searched using the software MrBayes 3.2.6 (Ronquist *et al.*, 2012). Posterior probabilities (PP) were calculated at the conclusion of the Markov-Chain-Monte-Carlo analysis and a traditional burn-in on the first 25% of the trees was performed. The Bayesian topology was visualized using the FigTree v1.3.1.program (Rambaut, 2009). The ML trees were reconstructed using the MEGA program package, version 5 (Tamura *et al.*, 2011). The robustness of the phylogenetic trees was estimated via bootstrap analysis using 1000 replications.

**Figure 3:**
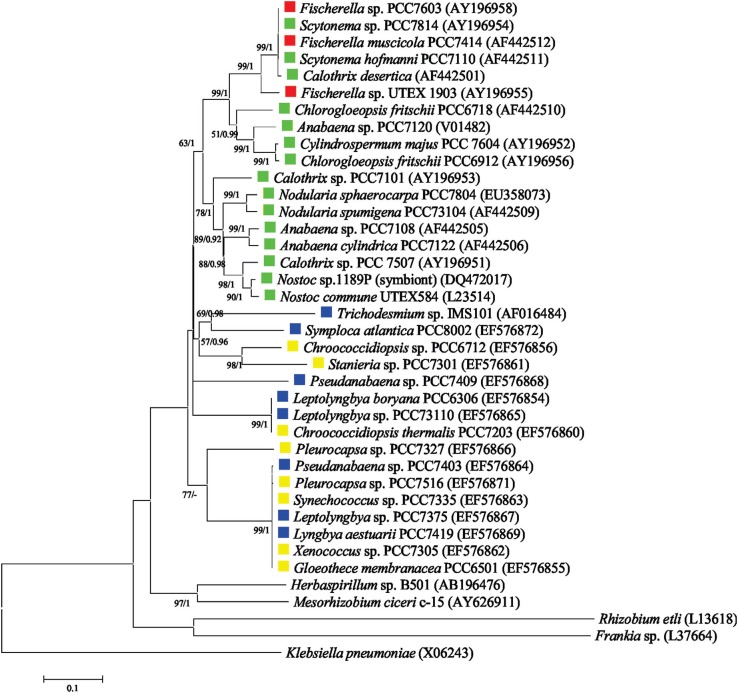
Maximum Likelihood (ML) phylogenetic reconstruction based on partial *nif*D gene sequences. A total of 39 nucleotide sequences of α-subunit of dinitrogenase were used. A matrix with 6,904 base pair lenght was obtained after alingment. The general time reversible evolutionary model of substitution with gamma distribution and with an estimate of proportion of invariable sites (GTR + G + I) was selected as the fittest for the alignment by MrModelTest 2.3 (Nylander, 2004). Phylogenetic trees were reconstructed using the ML and Bayesian methods. For Bayesian analysis, the trees were searched using the software MrBayes 3.2.6 (Ronquist *et al.*, 2012) and the Bayesian analysis consisted of two independent runs, with four Markov chains each, of 50 million generations sampled every 5,000 generations. Posterior probabilities (PP) were calculated at the conclusion of the Markov-Chain-Monte-Carlo analysis and a traditional burn-in on the first 25% of the trees was performed. The ML trees were reconstructed using the MEGA program package, version 5 (Tamura *et al.*, 2011). The robustness of the phylogenetic trees was estimated via bootstrap analysis using 1,000 replications. ML and Bayesian methods resulted in nearly identical topologies, with indications of bootstrap values (ML) and Bayesian PPs in the relevant nodes. The cyanobacterial morphologies are highlighted with different colours: yellow for unicellular strains, blue for filamentous non-heterocytous strains, green for filamentous heterocytous strains without branching, and red for filamentous heterocytous strains with true branching. Sequence data from this article can be found in the NCBI database under the accession numbers, which are presented together with the strain name.

**Figure 4:**
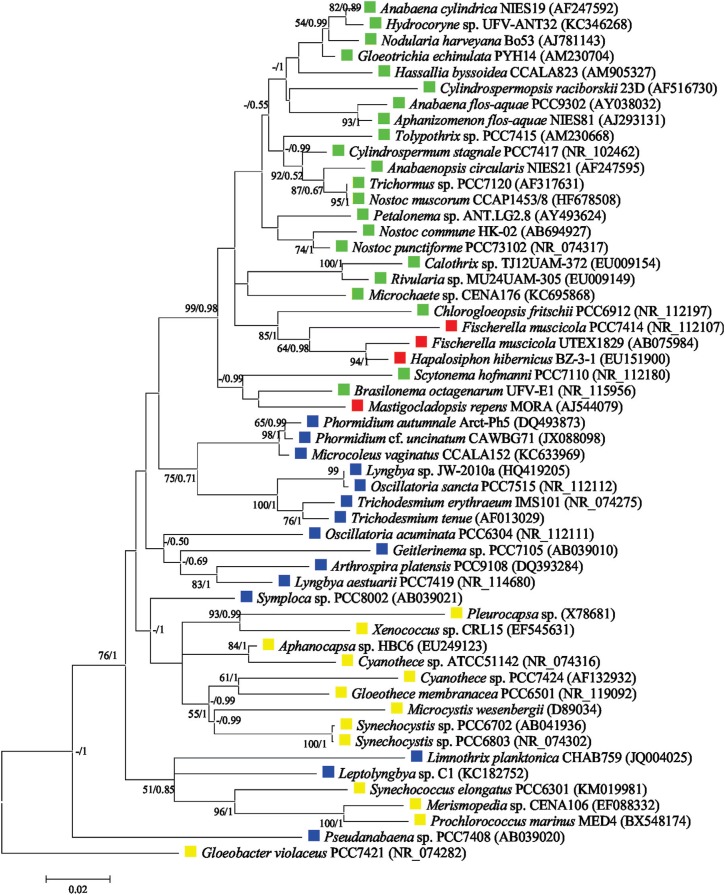
Maximum Likelihood (ML) phylogenetic reconstruction based on partial 16S rRNA sequences. A total of 54 sequences were used. A matrix with 1,463 base pair lenght was obtained after alingment. The general time reversible evolutionary model of substitution with gamma distribution and with an estimate of proportion of invariable sites (GTR + G + I) was selected as the fittest for the alignment by MrModelTest 2.3 (Nylander, 2004). Phylogenetic trees were reconstructed using the ML and Bayesian methods. For Bayesian analysis, the trees were searched using the software MrBayes 3.2.6 (Ronquist *et al.*, 2012) and the Bayesian analysis consisted of two independent runs, with four Markov chains each, of 50 million generations sampled every 5,000 generations. Posterior probabilities (PP) were calculated at the conclusion of the Markov-Chain-Monte-Carlo analysis and a traditional burn-in on the first 25% of the trees was performed. The ML trees were reconstructed using the MEGA program package, version 5 (Tamura *et al.*, 2011). The robustness of the phylogenetic trees was estimated via bootstrap analysis using 1000 replications. ML and Bayesian methods resulted in nearly identical topologies, with indications of bootstrap values (ML) and Bayesian PPs in the relevant nodes. The cyanobacterial morphologies are highlighted with different colours: yellow for unicellular strains, blue for filamentous non-heterocytous strains, green for filamentous heterocytous strains without branching, and red for filamentous heterocytous strains with true branching. Sequence data from this article can be found in the NCBI database under the accession numbers, which are presented together with the strain name.

Results obtained using *nif*D gene sequences support the monophyly of cyanobacteria, with a group of Proteobacteria as a sister group and a *Klebsiella* sequence as root (Figure 3). Indeed, previous analyses of the catalytic subunits of this enzyme complex indicate that the enzyme existed prior to the oxygenation of Earth's atmosphere (Latysheva *et al.*, 2012). Our phylogenetic analyses also demonstrated that the cyanobacterial *nif*D sequences group in a very similar way to the 16S rRNA topology, supporting a vertical ancestry of N_2_ fixation among cyanobacteria (Figures 3 and 4). The heterocytous cyanobacteria form a monophyletic lineage, with true branching cyanobacteria placed within the non-branching cyanobacteria group (Figure 3), indicating that the *nif*D gene from heterocytous strains share a common ancestor (Figure 3). Based on *nif*D gene sequences, cyanobacterial morphotypes belonging to Chroococcales and Synechococcales (unicellular), and Oscillatoriales/Pseudanabaenales (filamentous non-heterocytous) constitute polyphyletic groups (Figure 3). Taken together, these data indicate that morphological features and 16S rRNA phylogeny data are highly correlated with the evolutionary history of the *nif*D gene (Figures 1, 3 and 4), at least for this dataset, and mainly based on order level. It is important to mention however, that the monophyletic origin of *nif*D cannot be directly associated with morphological aspects. The phylogenetic reconstruction based on 16S rRNA sequences suggest that all heterocytous cyanobacteria (Nostocales) form a single monophyletic group (Henson *et al.*, 2004a; Tomitani *et al.*, 2006; Shih *et al.*, 2013) and support the polyphyly of true branching cyanobacteria (Figure 4). In addition, our analysis corroborates other molecular data from 16S rRNA sequences that demonstrate a polyphyletic origin of the unicellular and filamentous homocytous strains (Figure 4) (Litvaitis, 2002; Valério *et al.*, 2009; Andreote *et al.*, 2014; Silva *et al.*, 2014).

## The importance of an evolutionary pressure for N_2_ fixation

Careful phylogenetic analyses using 57 *nif*D nucleotide sequences and inferred amino acids sequences (Henson *et al.*, 2004b), and also proteins sets from 49 cyanobacterial genomes (Latysheva *et al.*, 2012), suggest the presence of a nitrogen fixing cyanobacteria common ancestor. This implies that N_2_-fixation genes had arisen approximately 3 billion years ago (Latysheva *et al.*, 2012) and lends support for the results observed in Figure 3. It has been assumed that fixed nitrogen was a limiting resource in the early Earth environment (Raven and Yin, 1998; Kasting and Siefert, 2001), once a decrease in the atmospheric CO_2_ concentration in the early Archaean (~3.5 billion years ago) might have entailed in a small availability of reduced nitrogen forms, synthesized from N_2_ and CO_2_ (Navarro-Gonzalez *et al.*, 2001). In addition, in this period, the Earth's reduced atmosphere might have allowed fixed nitrogen compounds to be stable (Kasting and Siefert, 2001, 2002). Altogether, these conditions, probably, were able to favour an evolutionary pressure for the establishment of a biological N_2_ fixation process at an early stage of prokaryotic evolution (Towe, 2002). Assuming that the origin of nitrogenase pre-dates the origin of N_2_ fixation, it is likely that respiratory enzymes or cyanide detoxification centers had been involved in this process (Fani *et al.*, 2000). Our data indicate that all cyanobacteria strains able to fix N_2_ are forming a monophyletic group when compared with the other, paraphyletic eubacteria group, suggesting that the intrinsic ability to fix N_2_ in cyanobacteria was most likely obtained directly from a common ancestor (Figure 3). Information from phylogenetic reconstructions and also the presence of *nif* genes in many groups of archaea and bacteria suggest that nitrogenase had already evolved within the last common ancestor (LCA) (Normand *et al.*, 1992; Fani *et al.*, 2000). However, it remains unclear whether the present distribution of *nif* genes in cyanobacteria has been obtained by horizontal gene transfer (HGT), or whether vertical descent had a larger impact on this process (Henson *et al.*, 2004b; Latysheva *et al.*, 2012). In the LCA hypothesis, the loss of nitrogenase genes by some groups reflects the modern scattered distribution of these among both Archaea and Bacteria, but not in all phyla, and neither in eukaryotes (Raymond *et al.*, 2004; Latysheva *et al.*, 2012). Meanwhile, HGT and genetic duplication events could happened between and within prokaryotic lines, helping to explain the presence of more than one nitrogenase gene copy in some bacteria (Kechris *et al.*, 2006). These copies could be related to a new nitrogenase family, which presents a different metal co-factor (Fe or V nitrogenases) (Thiel, 1993; Pratte *et al.*, 2006), showing that HGT is most likely a source of genetic diversity in cyanobacteria (Mulkidjanian *et al.*, 2006).

Given the crucial importance of nitrogenase for cyanobacterial N_2_ fixation, it is not surprising that alternative nitrogenases have been found (Eady, 1996; Masukawa *et al.*, 2009). Cyanobacteria have co-evolved during the course of planetary evolution and was already present when the change of oxidation state of both ocean and atmosphere occurred (Berman-Frank *et al.*, 2003). The low oxygen concentration in the early Earth might have acted as a selective pressure on nitrogenase, once information from paleosols indicates a high availability of reduced Fe rather than Mo. Then, a nitrogenase able to use Fe as a metal-cofactor would have been in great advantage (Anbar and Knoll, 2002). Accordingly, some physiological evidence has been presented for the existence of a Fe-nitrogenase in *Anabaena variabilis* (Kentemich *et al.*, 1991). In addition, a V-nitrogenase has been found only in the genera *Anabaena* (*A. variabilis* and *A. azotica*) and *Nostoc* (Kentemich *et al.*, 1988; Thiel, 1993; Masukawa *et al.*, 2009). The divergence between the Fe-dependent and V-dependent nitrogenases most likely occurred subsequently in the evolutionary history, and it is therefore reasonable to suggest that an ancestral NifD homolog might have had lower specificity with respect to its metal cofactor (Raymond *et al.*, 2004). The cyanobacterial photosynthesis led to a progressive increase of the atmospheric oxygen concentration in the Precambrian Earth, affecting negatively Fe availability. On the other hand, soluble oxidized Mo started to become more available in the oceans (Hofmann, 1976; Bekker *et al.*, 2004; Frei *et al.*, 2009). Thus, nitrogenase would be responsive for the environmental availability of V, Fe, and Mo that fluctuated with the changing redox state that characterised the Proterozoic Earth between 1 and 2 billion years ago (Normand and Bousquet, 1989; Anbar and Knoll, 2002). Furthermore, in addition to the higher availability of Mo, an increased efficiency of Mo-nitrogenase, compared with both V- and Fe-dependent enzymes, could also act as an additional selection pressure factor for the establishment of the Mo-dependent nitrogenase (Raymond *et al.*, 2004). Growth rates registered on V and Mo cultures of *A. variabilis* were essentially the same, although the catalytic efficiency of the alternative nitrogenase was lower than the one presented by the MoFe-nitrogenase (Kentemich *et al.*, 1988). In addition, the specific activity of the VFe-nitrogenase, at 30 °C, in *Azotobacter* is approximately 1.5 times lower than that of MoFe-nitrogenase (Miller and Eady, 1988).

## Hydrogen biosynthesis and strategies to improve hydrogen production in cyanobacteria

Molecular hydrogen was an essential source of energy during the early stages of the Earth, but lost its importance with the evolution of the photosynthetic machinery, that was able to use light more efficiently (Esper *et al.*, 2006). In cyanobacteria and other N_2_-fixing prokaryotes, H_2_ is synthesized as a by-product of nitrogenase during the N_2_ fixation process, and in a next step reaction, it may be oxidized by a hydrogenase (Berman-Frank *et al.*, 2003). Accordingly, in addition to nitrogenase, cyanobacteria may possess different enzymes related with H_2_ metabolism: an uptake hydrogenase which catalyses H_2_ consumption, and a bidirectional hydrogenase able to catalyse both H_2_ synthesis and oxidation (Tamagnini *et al.*, 2002). The presence of a bidirectional hydrogenase in cyanobacteria is unrelated with its capacity to fix N_2_ (Serebriakova *et al.*, 1994; Carrieri *et al.*, 2011). On the other hand, an uptake hydrogenase has been found in almost all the N_2_-fixing cyanobacteria examined thus far, with one reported exception - *Synechococcus* sp. BG 043511 (Ludwig *et al.*, 2006). The recycling of H_2_, by hydrogenases, is an important metabolic process, once it generates ATP and reduction equivalents, and provides an anoxic environment to nitrogenase activity (Bothe *et al.*, 2010). As important enzymes in the energy metabolism of microorganisms, hydrogenases are widespread in prokaryotes. The distribution and function of these enzymes has been expertly investigated elsewhere (Ludwig *et al.*, 2006; Barz *et al.*, 2010; Skizim *et al.*, 2012), once H_2_ is commonly considered as the future of “clean” energy (Dismukes *et al.*, 2008; Quintana *et al.*, 2011). Its combustion, different of fossil fuels, releases water as a product together with high amounts of energy which can be transformed in electricity (Dutta *et al.*, 2005). Furthermore, H_2_ is an unlimited energy source, and even with the lower efficiency of photobiological systems compared with electrochemical H_2_ production, this alternative shows economic viability due to the low production cost (Block and Melody, 1992; Lindblad, 1999; Dutta *et al.*, 2005). Notably, H_2_ production was registered for at least 14 cyanobacterial genera under a vast range of culture growth conditions (Tamagnini *et al.*, 2002), and although both the nitrogenase(s) and the bidirectional hydrogenase are capable of H_2_ production (Tamagnini *et al.*, 2000), it is reasonable to assume nitrogenase as a key enzyme for cyanobacterial H_2_ production (Kumazawa and Mitsui, 1994; Yoshino *et al.*, 2007; Sakurai *et al.*, 2015). As discussed above, alternative nitrogenases exhibit lower catalytic activities compared with MoFe-nitrogenase (Kentemich *et al.*, 1988; Miller and Eady, 1988), and might not be assumed as candidates for H_2_ production (Hallenbeck and Benemann, 2002).

Cyanobacterial photohydrogen production has been already carried out with N_2_-fixing strains (Lichtl *et al.*, 1997; Tsygankov *et al.*, 1997, 1998) in which the net H_2_ production is the result between the H_2_ evolution catalyzed by nitrogenase and H_2_ consumption catalyzed by the uptake hydrogenase. The inactivation of [NiFe]-uptake hydrogenase in N_2_-fixing cyanobacteria leads to an efficient increase in the H_2_ produced: 3 to 7 x-fold more than in wild-type cells under optimal conditions (Masukawa *et al.*, 2002; Yoshino *et al.*, 2007). On the other hand, due to the sensitivity of nitrogenase and hydrogenases to O_2_ (Zehr *et al.*, 1993; Serebryakova *et al.*, 1996; Tamagnini *et al.*, 2002), strategies such as biophotovoltaic cells (BPVs) and anaerobic growth conditions have been tested to separate the O_2_ for H_2_ production, in an attempt to improve the yield of this process (Bombelli *et al.*, 2011; Bradley *et al.*, 2013). However, it seems reasonable to assume that another possible solution to this issue is the use of heterocytous cyanobacteria. These organisms appear as an interesting solution given that they present at least two different cell types (vegetative cells and heterocytes), where the O_2_ and H_2_ evolving activities occur spatially separated.

Many cyanobacteria are facultative anaerobes that can produce H_2_ as a by-product of the dark anoxic catabolism of photosynthetic compounds, mainly glycogen (Stal and Moezelaar, 1997; Das and Veziroglu, 2001; McNeely *et al.*, 2010). It was shown that after hydrogenase activation by anaerobic conditions in the dark, the amount of H_2_ produced at light conditions by an engineered strain of *Synechocystis* lacking the quinol and cytochrome *c* oxidase (Gutthann *et al.*, 2007) increased 12 fold compared to wild-type cells (Gutthann *et al.*, 2007). Furthermore, the disruption of the nitrate assimilation pathway produced from 10 to 140 fold more H_2_ (Baebprasert *et al.*, 2011), and cells supplemented with ammonium, as the nitrogen source, evolved about twofold more H_2_ than cells grown with nitrate (Baebprasert *et al.*, 2011). Notably, increasing concentrations of nickel (Ni) during cell growth seem to be clearly important for H_2_ production. Thus, NiCl_2_ supplementation in *Arthrospira maxima* kept under low light increases H_2_ production following anaerobic induction in darkness. Additionally, Ni supplemented cultures evolve H_2_ at initial rates 18 fold higher than unsupplemented ones (Carrieri *et al.*, 2008). Collectively these results indicate that both metabolic engineering and growth conditions will have clear impacts on H_2_ production and therefore, further combined studies are required to increase our knowledge on this important cyanobacterial topic.

Although the H_2_ production in N_2_-fixing cyanobacteria has been extensively investigated (Kumazawa and Mitsui, 1994; Serebriakova *et al.*, 1994; Kumazawa and Asakawa, 1995; Tsygankov *et al.*, 1997, 1998; Borodin *et al.*, 2000; Masukawa *et al.*, 2002; Yoshino *et al.*, 2007), a focus on non-fixing cyanobacteria strains, mainly *Synecochystis* sp. PCC6803, has also recently appeared (Gutthann *et al.*, 2007; Baebprasert *et al.*, 2011; McCormick *et al.*, 2013). The availability of its genomic sequence coupled with the acquired ability to be naturally transformable has clearly promoted the usage of this strain. However, recent evidence suggests that *Synechococcus* sp.WH5701, a N_2_-fixing cyanobacteria, may have a higher capacity for extracellular electron transport in comparison to *Synechocystis* (McCormick *et al.*, 2011). Thus, it seems likely that analysing the yield of N_2_-fixing cyanobacteria in BPVs and in other conditions mentioned here and compare it with *Synechocystis*, mainly those heterocytous strains with inactivated [NiFe]-uptake hydrogenase (Masukawa *et al.*, 2002; Yoshino *et al.*, 2007), might provide an interesting research avenue to be persued. In addition, it should be kept in mind that, although extensive efforts have been made to produce H_2_ from cyanobacteria, this approach appears to be still in a very early stage of development (Akkerman *et al.*, 2002; Wijffels *et al.*, 2013), and therefore, a number of technological aspects, such as the cost of nutrients and bioreactors, should be considered during the design of future plans for photobiological H_2_ production (Sakurai *et al.*, 2013, 2015). Although, significant challenges remain in the potential developing of cyanobacteria for biological H_2_ production, we hope that through the use of the above discussed targets, subsequent studies will increase our knowledge and bring us closer to realizing the biotechnological potential of nitrogenase-mediated H_2_ production by these microorganisms.

## Concluding remarks

Despite the relationships observed here between the *nif*D sequences with regard to both morphological and molecular (16S rRNA) relationships previously observed in the cyanobacterial group, many open questions remain about cyanobacterial evolution and metabolism. It is reasonable to assume that the different strategies observed in cyanobacteria (spatial and temporal separation) to improve N_2_ fixation were associated with small alterations in the *nif* nucleotide sequences, despite large changes in morphology. Our future ability to answer these questions is dependent on fundamental work providing a fuller understanding of these processes and on how they are regulated. Although many biological and technological challenges need to be overcome, we believe that improvement of the N_2_ fixation process will be directly associated with H_2_ production as one of the leading contenders for renewable energy. It should also be kept in mind that the development of large-scale and economical photobiological H_2_ production, which might be linked to improved cyanobacterial N_2_ fixation, most likely will make meaningful contributions to mitigate climate change and also provide new employment opportunities, particularly in areas unsuitable for modern agriculture.
